# Human umbilical cord mesenchymal stem cell transplantation restores damaged ovaries

**DOI:** 10.1111/jcmm.12571

**Published:** 2015-04-29

**Authors:** Shao-Fang Zhu, Hong-Bo Hu, Hong-Yan Xu, Xia-Fei Fu, Dong-Xian Peng, Wei-Yan Su, Yuan-Li He

**Affiliations:** 1 Medical College of Shaoguan UniversityShaoguan, GuangDong, China; 2 Department of Obstetrics and Gynaecology, Zhujiang Hospital, Southern Medical UniversityGuangzhou, GuangDong, China; 3 Department of Obstetrics and Gynaecology, The Yuebei People’s Hospital of ShaoguanShaoguan, GuangDong, China

**Keywords:** umbilical cord, human umbilical cord mesenchymal stem cell, ovarian function

## Abstract

Ovarian injury because of chemotherapy can decrease the levels of sexual hormones and potentia generandi of patients, thereby greatly reducing quality of life. The goal of this study was to investigate which transplantation method for human umbilical cord mesenchymal stem cells (HUMSCs) can recover ovarian function that has been damaged by chemotherapy. A rat model of ovarian injury was established using an intraperitoneal injection of cyclophosphamide. Membrane-labelled HUMSCs were subsequently injected directly into ovary tissue or tail vein. The distribution of fluorescently labelled HUMSCs, estrous cycle, sexual hormone levels, and potentia generandi of treated and control rats were then examined. HUMSCs injected into the ovary only distributed to the ovary and uterus, while HUMSCs injected *via* tail vein were detected in the ovary, uterus, kidney, liver and lung. The estrous cycle, levels of sex hormones and potentia generandi of the treated rats were also recovered to a certain degree. Moreover, in some transplanted rats, fertility was restored and their offspring developed normally. While ovary injection could recover ovarian function faster, both methods produced similar results in the later stages of observation. Therefore, our results suggest that transplantation of HUMSCs by tail vein injection represents a minimally invasive and effective treatment method for ovarian injury.

## Introduction

Protection of ovary function and the repair of injured ovary tissue represent key concerns both before and after chemotherapy treatments 1–4. In recent years, hormone replacement therapy, cryopreservation techniques, assisted reproductive technology and gonadotropin-releasing hormone agonist (GnRHa) treatments have been used to prevent and treat premature ovarian failure induced by chemotherapy 5–7. However, there are disadvantages associated with each of these methods. For example, long-term hormone replacement therapy may increase the risk of breast cancer or other diseases 8. Ethical and legal controversies are associated with oocyte donation for assisted reproduction technologies and embryo transfer, particularly regarding the age and indications of oocyte recipients and the rights and interests of the resulting children 9. The cost of cryopreserved ovarian technology is high. Finally, GnRHa treatments have shown no therapeutic value for the treatment of serious premature ovarian failure in all cases 10. Therefore, the opportunity to recover normal ovarian function would represent an important advance in reproductive biology.

In our previous study, we investigated the capacity for bone marrow-derived mesenchymal stem cells (BMSCs) to treat premature ovarian failure 11. BMSCs were injected into rat models of premature ovarian failure incurred by chemotherapy, and the number of follicles at all levels in these rats’ ovary increased. Levels of estradiol (E_2_) also increased, while levels of follicle-stimulating hormone (FSH) decreased, compared with the model group that did not undergo transplantation. BMSCs have also been shown to secrete VEGF, insulin-like growth factor 1 (IGF-1) and hepatocyte growth factor (HGF), as well as other factors, and to inhibit ovarian granulosa cell apoptosis. Taken together, these results indicate that BMSCs have the potential to repair ovary structure and improve ovarian endocrine function. However, the fertility of the rats and the health of their offspring following cell transplantation have not been examined. In addition, MSCs derived from adult tissues, such as bone marrow, fat, liver and skin, have many disadvantages. For example, there is an insufficient supply of stem cells from BMSCs, BMSCs exhibit decreased proliferation and a reduced differentiation capacity with age 12, and BMSCs are associated with a risk of infection and the development of tumours following stem cell transplantation 13,14. Furthermore, it has been shown that bone marrow-derived MSCs (BMSCs) can cause secondary damage in patients following transplantation 15.

Alternatively, collecting human umbilical cord mesenchymal stem cells (HUMSCs) extracted from Wharton’s jelly of the umbilical cord is convenient is an ideal seed cell for clinical treatment with lower oncogenicity, and is associated with a lower contamination rate by viruses and bacteria compared to BMSCs. Furthermore, HUMSCs do not express immunological rejection-related markers, resulting in the absence, or very minimal chance, of immunological rejection in an allograft. The ethics associated with this approach are also much less controversial 16.

In the present study, the treatment effects associated with the administration of HUMSCs *via* ovary injection or tail vein injection were compared. In addition, the fertility of the rats after HUMSC transplantation was evaluated, as well as the health of the resulting offspring.

## Materials and methods

### Experimental animals

Female Wistar rats (SPF class, 180–200 g, 7-week old) were purchased from the Animal Experimental Center, Zhongshan Medical University. Breeding conditions were maintained at a temperature of 30 ± 2°C, with a 14 hrs/10 hrs light/dark cycle. Vaginal fluid was collected daily at 8:00 am to monitor estrous cycles, and only rats with normal estrous cycles were used. Prior to experiments, 1 ml of blood was collected from each rat during interphase of a normal estrous cycle. Blood samples were centrifuged for 10 min at 3000 rpm and the resulting sera were stored at −80°C. Basal levels of rat E_2_ and FSH were measured for each serum sample. The Southern Medical University institutional review committee approved all of the experiments performed involving animals and human cells.

### Experimental design

HUMSCs were initially isolated from patients who underwent pelvic stenosis by caesarean section primipara. The biological characteristics of these cells were examined to evaluate the success of our cell isolation technique. To evaluate the immune response in the rats following HUMSC transplantation, HUMSCs were first transplanted into normal rats, and then the resulting innate immune response and transplant cell survival rates were examined. Based on these data, HUMSCs were transplanted into ovary damaged rats *via* ovary injection and tail vein injection respectively. The resulting recovery of ovary function was evaluated using physiology tests, histology tests and reproduction tests. The details of these assays are described as follows.

### Separation, collection and identification of HUMSCs

Two patients (aged 24 and 26 years) underwent pelvic stenosis by caesarean section primipara. Except for a contracted pelvis, there were no other obstetric complications and the patients had no history of infectious diseases. The neonates were healthy full-term infants and the sample collection process and prior test consent were described and approved by the pregnant women and their families. The hospital Ethics Committee also approved the experiments performed.

Umbilical cords from the healthy caesarean deliveries were collected and washed with PBS to remove the residual blood. Both arteries and veins were removed. The cleaned umbilical cords were then cut into 1 cm pieces, homogenized to a volume of 1–2 mm^3^, and put into (DMEM)-F12 culture medium (cat. no. SH30023.01B; Hyclone, Logan, Utah, US) with 10% foetal bovine serum (FBS; Hyclone, Logan, Utah, US). Cells were cultured with 5% CO_2_ at 37°C. After the first subculture, cells were passaged with a 1:3 ratio every 3 days. Second passage HUMSCs were used for experiments.

Secondary passages of HUMSCs were trypsinized and dissociated into single cell suspensions. Monoclonal antibodies (Pharmingen, San Diego, CA, USA) that recognize CD29, CD44, CD73, CD105, CD90, CD14, CD34, CD45, CD106, CD133, HLA-I and HLA-DR (at a concentration of 20 μl/10^6^ cells) were used. Mouse IgG was used as a negative control. Cells were incubated with antibodies (5 μl each) for 30 min at 4°C. Antibody binding was detected using flow cytometry (FACS Calibur; BD Biosciences, San Jose, CA, USA).

### Detection of cell growth, apoptosis and cell cycle for HUMSCs

For cell growth assays, cells were cultured in 96-well plates (1000 cells/100 μl), with five wells plated for each sample. Cell growth was measured for 7 continuous days. Briefly, 10 μl of CCK-8 solution (Beyotime Institute of Biotechnology, Jiangsu, China) was added to each well. After plates were incubated for 1 hr at 37°C, absorbance values at 450 nm (A_450nm_) were measured and recorded using a spectrophotometer. Cell growth curves were generated, with medium alone used as a blank control.

For apoptosis assays, aliquots of each cell suspension (100 μl) were placed in 5 ml tubes and were incubated with 5 μl of Annexin V/FITC (BD Biosciences) and 10 μl of propidium iodide (PI, 20 μg/ml) in the dark. After 15 min, 400 μl PBS was added to each tube and the cells were analysed by flow cytometry.

For cell cycle analysis, the cells were digested with trypsin, resuspended to 1 × 10^6^ cells/ml and were centrifuged (1000 rpm, 5 min). The resulting cell pellets were fixed with 75% ethanol at −20°C. Prior to analysis, the cells were centrifuged (1000 rpm, 5 min), RNase was added (10 μg/ml) to eliminate RNA contamination, and cells were treated with PI at 4°C in the dark. After 30 min, the samples were analysed by flow cytometry.

### Measuring secreted VEGF, IGF-1 and HGF

Secondary passages of HUMSCs were cultured in flasks (5 × 10^5^ cells/flask) and grown to confluence. Cells were then cultured in complete culture medium (DMEM supplemented with 10% FBS) for an additional 24 hrs, before the culture medium was collected and centrifuged. ELISAs (R&D Systems, Minneapolis, MN, USA) were used to detect levels of VEGF, IGF-1 and HGF in supernatant samples, with culture medium serving as a negative control 11.

### Labelling of HUMSCs with PKH26

Cells were incubated with a fluorescently labelled, cell membrane-binding molecule, PKH26 (Sigma-Aldrich Co, St. Louis, MO, USA), as previously described 17. Briefly, a total of 2 × 10^7^ cells (passage 2) were washed and resuspended in serum-free DMEM. Following centrifugation at 400 rpm for 5 min, the supernatant was discarded. The cells were gently resuspended and completely dissolved in 1 ml of Solution C (dilutant for PKH26) (Sigma-Aldrich Co). The cells were immediately combined with 4 × 10^−6^ M PKH26 staining reagent (diluted in Solution C) and were incubated at 25°C with gentle rocking. After 2–5 min, staining activity was inhibited with the addition of the same volume of serum for 1 min. The cells were centrifuged at 400 rpm for 10 min at 25°C, and the supernatant was removed. The cells were transferred to a new tube and were washed three times with serum-free DMEM. Of complete culture medium, 10 ml was then added, and the cells were centrifuged and adjusted to an appropriate density for observation under a fluorescence microscope.

### Immune response following HUMSC transplantation

Three groups of five rats (SPF class, 180–200 g, 7-week old) with normal estrous cycles were established. The first group was the control group and these rats did not receive HUMSC transplantation. The second group was the ovary injection group and the third group was the tail vein injection group. The latter two groups underwent successful transplantations of PKH26-labelled HUMSCs. The transplantation procedure used is described in a separate section below. Following the transplantation of HUMSCs, rats were killed 1, 15, 30 and 45 days later. Brain, liver, kidney, urocyst, ovary, uterus and other organs were immediately frozen and sectioned. The distribution of HUMSCs within these sections was analysed using fluorescence microscopy. Sections were also subjected to haematoxylin and eosin staining to analyse pathological changes. Comparisons were made between organs.

### Induction of ovary damage

Rats with normal estrous cycles were divided into four groups (with 25 rats per group), namely control group (normal control), model group (chemotherapy-induced ovarian damage), ovary injection group and tail vein injection group. In the latter three groups, the rats received a loading dose of cyclophosphamide (CTX; 50 mg/kg) followed by daily intraperitoneal CTX injection at 8 mg/kg for 14 consecutive days, to establish rat models of chemotherapy-induced ovarian damage. Immediately after the last injection of CTX 11, PKH26-labelled cells were injected into the tail vein injection group and the ovary injection group. For the tail vein injections, PKH26-labelled cells were suspended in physiological saline at a concentration of 1 × 10^6^ cells/ml. To prevent cell clusters that could induce an embolism, fully discrete cells were confirmed under light microscopy prior to injection. For the injections, rats were placed in a rat holder (Beijing Ji Tai, Ltd, Beijing, China) and the tail was wiped with alcohol. Warm water or a warm towel was used to induce vein dilation prior to injection of 1 ml HUMSCs into the tail vein with a 1 ml syringe (Fig. S1A).

For the ovary injections, PKH26-labelled cells were suspended in a physiological saline solution at a concentration of 5 × 10^7^ cells/ml. The rats to be injected received an intraperitoneal injection of 10% chloral hydrate for anaesthesia, and then were fixed in a supine position. Iodine and alcohol disinfection was applied before an incision was made in the abdomen. After the ovary was found, 20 μl of HUMSCs were injected into the ovarian tissue (Fig. S1B) 11. To prevent post-operative infections, each rat in the ovary transplantation group received 100,000 U penicillin for 3 days.

In our previous studies, no statistically significant differences were detected between rats receiving injections of physiological saline into ovary tissue *versus* an untreated normal group. Therefore, a saline control group was not established in the current study. For the model group, these rats did not receive further treatment.

### Levels of sex hormones and estrous cycle monitoring

Prior to the induction of ovary damage, blood samples were drawn from the tails of rats in diestrus, and these provided basal hormone levels. Blood samples were also drawn from rat tails 1, 15, 30, 45 and 75 days after the induction of ovary damage was complete. The samples were incubated overnight at 4°C, then were centrifuged for 10 min at 3000 rpm. The resulting supernatant sera were collected and stored at −80°C. Levels of E_2_ and FSH were measured by ELISA. Vaginal fluid was collected at 8 a.m. each morning to observe changes in the estrous cycle before and after transplantation.

### Ovarian morphology and follicle counting

At various time-points following transplantation (*e.g*. 1, 15, 30 and 45 days), five rats from each group were randomly selected and killed. Ovarian specimens were subsequently collected and frozen sections of these samples were stained with haematoxylin and eosin. The ovary structure for each sample was observed using a light microscope. In addition, various classes of follicles were counted 11. A primordial follicle refers to granule cells surrounding a single fusiform oocyte (Tables1–4). A primary follicle is surrounded by at least three granule cells, resulting in a cubic shape. A secondary follicle is surrounded by at least two layers of granulosa cells, yet has no follicular cavity. Antral follicles contain at least two granulosa cells and have a follicular cavity (Fig. S2).

**Table 1:** Comparison of the number of primordial follicles (*n* = 5, mean ± SD)

Group	1 days	15 days	30 days	45 days	*F*-value	*P*-value
Control	460.80 ± 36.56	446.40 ± 50.49	460.80 ± 40.31	462.40 ± 39.88	0.620	0.612
Model	423.40 ± 19.44	366.00 ± 18.69	330.20 ± 23.12	324.20 ± 14.65	28.088	<0.001
Ovary injection	442.80 ± 16.78	398.60 ± 9.84	391.60 ± 15.44*	390.20 ± 12.87*	15.896	<0.001
Tail vein injection	459.00 ± 16.69	392.40 ± 12.44	392.00 ± 18.10*	389.00 ± 11.98*	25.517	<0.001
*F*-value	2.661	7.121	20.896	47.055		
*P*-value	0.083	0.003	<0.001	<0.001		

* *P* < 0.05 model *versus* ovary injection and tail vein injection.

**Table 2:** Comparison of the number of primary follicles (*n* = 5, mean ± SD)

Group	1 days	15 days	30 days	45 days	*F*-value	*P*-value
Control	176.60 ± 15.60	190.20 ± 8.76	191.00 ± 5.15	192.80 ± 9.63	2.525	0.094
Model	161.00 ± 5.96	131.60 ± 6.88	95.80 ± 8.47	94.40 ± 10.04	79.765	<0.001
Ovary injection	176.80 ± 14.08	138.80 ± 12.11	133.80 ± 13.03*	135.60 ± 5.86*	15.270	<0.001
Tail vein injection	168.20 ± 6.61	149.00 ± 13.66	134.80 ± 13.39*	138.00 ± 7.81*	9.670	0.001
*F*-value	2.207	30.009	68.811	112.890		
*P*-value	0.127	<0.001	<0.001	<0.001		

* *P* < 0.05 model *versus* ovary injection and tail vein injection.

**Table 3:** Comparison of the number of secondary follicles (*n* = 5, mean ± SD)

Group	1 days	15 days	30 days	45 days	*F-*value	*P*-value
Control	74.00 ± 6.93	74.80 ± 6.34	75.40 ± 4.10	79.40 ± 2.08	1.056	0.395
Model	52.80 ± 6.61	43.40 ± 4.56	40.00 ± 2.92	35.00 ± 3.39	13.336	<0.001
Ovary injection	54.80 ± 6.18	48.20 ± 7.56	50.40 ± 3.44*	51.40 ± 2.60*	1.324	0.301
Tail vein injection	50.00 ± 7.42	50.40 ± 6.02	49.60 ± 5.81*	48.80 ± 2.59*	0.071	0.975
*F*-value	12.880	25.521	64.522	236.512		
*P*-value	<0.001	<0.001	<0.001	<0.001		

* *P* < 0.05 model *versus* ovary injection and tail vein injection.

**Table 4:** Comparison of the number of antral follicles (*n* = 5, mean ± SD)

Group	1 days	15 days	30 days	45 days	*F*-value	*P*-value
Control	83.20 ± 6.02	85.00 ± 4.74	84.80 ± 6.72	82.40 ± 5.41	0.238	0.869
Model	59.60 ± 4.45	45.60 ± 5.03	42.00 ± 4.58	43.20 ± 4.38	15.531	<0.001
Ovary injection	60.00 ± 5.70	59.60 ± 2.79*	53.00 ± 5.34*	57.80 ± 2.95*	2.663	0.083
Tail vein injection	59.60 ± 7.89	53.00 ± 5.05*	51.60 ± 5.92*	59.60 ± 2.07*	2.336	0.112
*F*-value	18.263	72.253	44.026	85.285		
*P*-value	<0.001	<0.001	<0.001	<0.001		

* *P* < 0.05 model *versus* ovary injection and tail vein injection.

### Observation of reproductive function

Five rats from each group were randomly selected for breeding 15 days after transplantation. Two female rats were housed with one male rat. The reproductive function was observed in the following 6 months.

### Development of young rats

The growth and development of offsprings were monitored. Bodyweight increments and sexual maturation of rats in the different groups were compared.

### Statistical analysis

SPSS 13.0 software (SPSS Inc, Chicago, IL,USA) was used for data analysis, and data are reported as the mean ± SD. Using a factorial anova test, data from multiple groups at different time-points were analysed. In addition, data for each time-point for multiple groups were analysed by one-way anova. A *P*-value less than 0.05 was considered statistically significant.

## Results

### Cell morphology and cell growth

Cultured, primary HUMSCs were adherent, and after 72 hrs in culture, cells exhibited fusiform shapes with fibroblast-like morphology. Abundant cytoplasm and large nuclei were also observed. Cells grew in parallel arrangements or with spiral growth, and cells at the vortex centre exhibited a multi-layer distribution pattern. With consistent passaging, the cells grew quickly and became confluent within 3–4 days (Fig. S3a).

### Analysis of immune phenotype

Flow cytometry detected expression of CD29, CD44, CD73, CD90, CD105 and HLA-I by cultured HUMSCs. In contrast, expression of endothelial cell antigen CD106, 133, haematopoietic stem cell antigen CD34, leucocyte common antigen CD45 and HLA-DR (MHC-II; a major white blood cell-related antigen) were not detected. In combination, these results suggest that HUMSCs are MSCs that express the mesenchymal cell-specific markers, CD29, CD44, CD73, CD90, CD105 and HLA-I, and they do not express the haematopoietic stem cell markers, CD14, CD34, CD45, or the endothelial cell-specific antigens, CD106 and CD133 (Fig. S3b).

### Cell proliferation, apoptosis and cell cycle progression

The proliferation of second passage HUMSCs was evaluated by adding cck-8 to the growth medium containing 10% FBS and no growth factors. The cells grew slowly for 1–2 days, and then grew rapidly over days 3–6. During this logarithmic growth phase, cell doubling times were 2.560 ± 0.117 days. Afterwards, a plateau in cell growth was observed (Fig. S4A).

HUMSCs were also stained with Annexin V/FITC and PI to detect cell apoptosis. Normal cells (80.26%), cells undergoing early apoptosis (1.39%) and late apoptosis (12.56%), as well as dead cells (5.80%) were detected (Fig. S4B).

Analysis of cell cycle progression with PI staining showed 67.48% of cultured HUMSCs were in the G0/G1 phase (with an abscissa peak value of 48.92), 32.52% were in the S phase (with an abscissa peak value of 96.84), and none of the cells were in the G2/M phase. Therefore, the G2/M% + S% value was 32.52%, suggesting that HUMSCs were undergoing active proliferation (Fig. S4C).

### Secretion of VEGF, IGF-1 and HGF

In cell culture medium without HUMSCs, VEGF, HGF or IGF-1 was not detected. In contrast, the supernatant of HUMSCs contained the following concentrations of HGF, VEGF and IGF: 68.292 ± 5.167 pg/ml, 6.633 ± 1.025 ng/ml and 67.238 ± 2.014 pg/ml, respectively.

### Results of PKH26 labelling

Staining of HUMSCs with PKH26 resulted in a consistent, clear and uniform distribution of labelled cell membranes that was observed using an inverted fluorescence microscope (Fig. S5 a1, a2). Moreover, cell survival rate following staining was 99% according to Trypan blue staining.

### Clinicopathological characteristics of the rat groups for the immune response following HUMSC transplantation

There were no differences in the feeding, movement or bowel relief observed for rats in the ovary injection group and the tail vein injection group *versus* the control group. In addition, there were no reports of somnolence, vomiting, convulsions, sudden death, anuria, abnormal mental state. The rats in the ovary injection group and the tail vein injection group also did not exhibit characteristics of expiratory dyspnoea, bleeding, moulting, hematuresis, bloody stool, or other acute and chronic immunological rejection responses. However, one rat in the tail vein injection group did exhibit abnormalities of adduction in the left arm, weak abduction in the lower limbs, a hard adductor and cerebral infarction 7 days after the injection of HUMSCs. Correspondingly, white infarct areas were observed in the right side of the brain cortex using TTC assays, and these were consistent with the hemiplegia observed (Fig. S6). No obvious lymphocytic infiltration or structural damage was observed in the ovary (Fig. S7, a1-a3), uterus (Fig. S7, b1-b3), liver (Fig. S7, c1-c3), kidney (Fig. S7, d1-d3) or brain (Fig. S7, e1-e3) tissue sections that were obtained from the ovary injection group and the intravenous injection group and were stained with haematoxylin and eosin. There were also no obvious differences between these sections and the sections obtained from the normal control group.

### HUMSCs transplantation improved ovarian function that was damaged by chemotherapy

#### Ovary morphology

**Figure 1:**
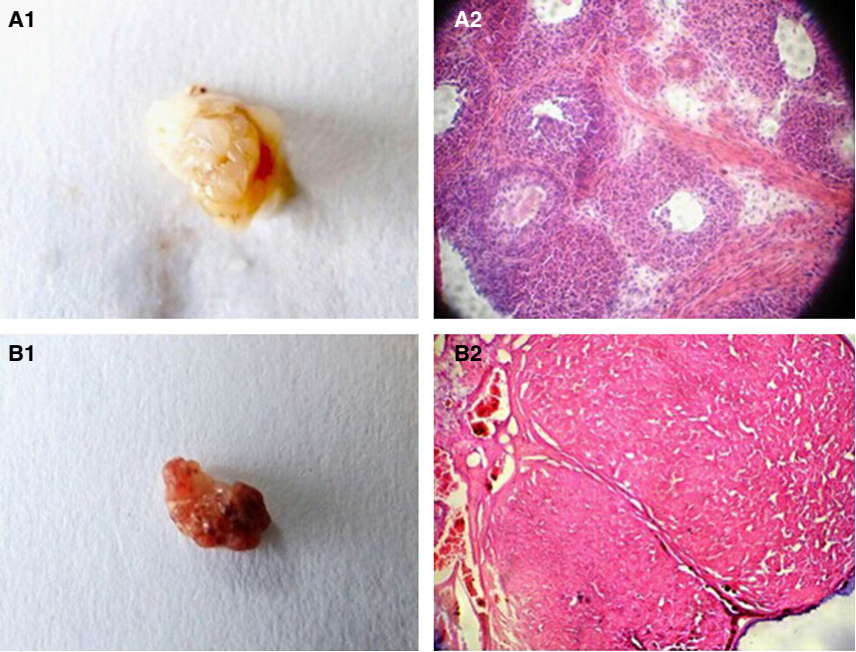
Resected ovaries from rats in the control group (A1) and the model group (B1). Corresponding pathological sections stained with haematoxylin and eosin are shown in (A2) and (B2) respectively.

In the control group, the ovaries had normal surfaces (Fig.1A1). However, following the injection of CTX, rat ovaries were found to narrow, they developed a semitransparent surface, and/or had a decrease in transparent uplifts (Fig.1B1). In addition, local hyperaemia was observed in some ovaries. Pathological studies and haematoxylin and eosin staining found the number of ovarian follicles to be markedly reduced (Fig.1A2 and B2), and an increase in bottoming was detected. In particular, severe fibrosis was observed (Fig.1B2). Taken together, these observations indicate that the ovary sustains an interstitial injury following the injection of CTX. For the ovary injection group and the tail vein injection group, no obvious change in the ovary surfaces was detected 15, 30 or 45 days after the injection of HUMSCs compared to the control group and the model group.

#### Distribution of labelled cells in the injected rats

Staining of HUMSCs with PKH26, a molecule that binds the cell membrane, allowed the injected cells to be monitored following transplantation. However, the intensity of the fluorescence gradually decreased with time. Labelled HUMSCs in the ovary injection group primarily localized to restricted regions of the ovary (Fig. S8, a1 and a2), with aggregation observed at the injection site. The regions of the ovary farther from the injection site contained fewer labelled HUMSCs, and none of the labelled HUMSCs were found in the ovarian follicle (Fig. S8, a1 and a2). Labelled HUMSCs were also found in some regions of the uterus (Fig. S8, b1 and b2), although they were not detected in other organs. In the tail vein injection group, labelled HUMSCs were found to be evenly distributed among the ovary (Fig. S9, a1 and a2), uterus (Fig. S9, b1 and b2), liver (Fig. S9, c1 and c2) and kidney cortex (Fig. S9, d1 and d2), yet were not found in brain tissues. In the ovary, labelled HUMSCs mainly localized to the stroma ovarii, and not to the ovarian follicle. Labelled HUMSCs were also detected in the uterus, mainly in the tunica muscularis, while some labelled HUMSCs were present in the intima. In liver tissue, labelled HUMSCs were evenly distributed, and clear outlines of all liver structures were observed, including the bile ducts and blood vessels. Furthermore, labelled HUMSCs were also observed in kidney tissues, especially the kidney cortex, and some renal corpuscles were clearly outlined.

#### Changes in estrous cycle

Rats in the control group exhibited normal estrous cycles throughout the observation phase as detected by vaginal smears. In contrast, dysfunctional estrous cycles, a decrease in estrous cycles and in some cases, an absence of estrous cycles were observed for rats in the model group, the ovary injection group and the tail vein injection group. Thus, it appears that injections of CTX damaged the rat estrous cycle. Three months after the injection of CTX, estrous cycles still had not recovered in the model group. However, normal estrous cycles were detected in one rat of the ovary injection group and in one rat of the tail vein injection group 45–90 days after the injection of CTX. Rats in the control group consistently maintained a normal estrous cycle.

**Figure 2:**
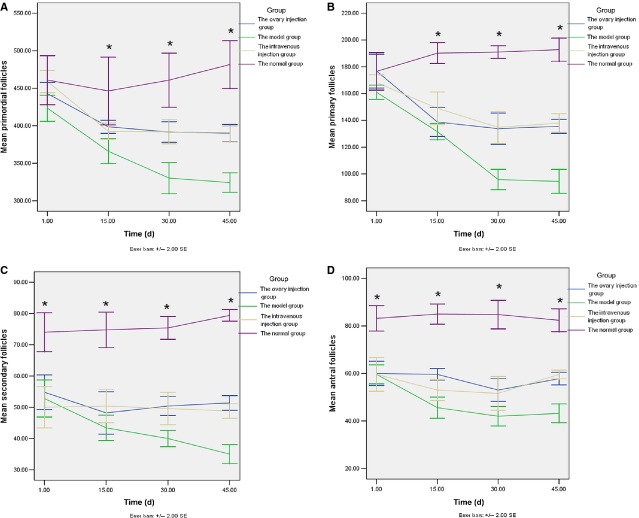
Number of primordial follicles (A), primary follicles (B), secondary follicles (C) and antral follicles (D) detected in the ovaries of rats in the various experimental groups as indicated (d = 0 indicates the day that the ovary damage model was established). At various time-points following transplantation (*e.g*. 1, 15, 30 and 45 days), five rats from each group were randomly selected and killed; *P < 0.05.

Pathological studies of the ovary tissues found no statistical difference in the number of primordial and primary follicles that were present the same day after injecting CTX for each group (Fig.2A and B). However, there was a significant decrease in the number of secondary follicles and sinus follicles present the same day when the rat models of chemotherapy-induced ovarian damage were establish compared with the control group. These results appear to represent the early effects of CTX-induced damage (Fig.2C and D). After 15 days, the number of primordial and primary follicles in the model group, the ovary injection group, and the tail vein injection group decreased compared with the control group (Fig.2A and B), while the number of secondary and antral follicles increased compared to the model group (Fig.2C and D). There was no difference in the number of secondary follicles observed for the ovary injection group and the tail vein injection group at any of the time-points assayed (Fig.2C). However, 15 days later, the number of antral follicles detected in the ovary injection group was higher than that for the tail vein injection group (Fig.2D). Thirty days later, this difference was not statistically significant (Fig.2D).

#### Levels of sex hormones

**Figure 3:**
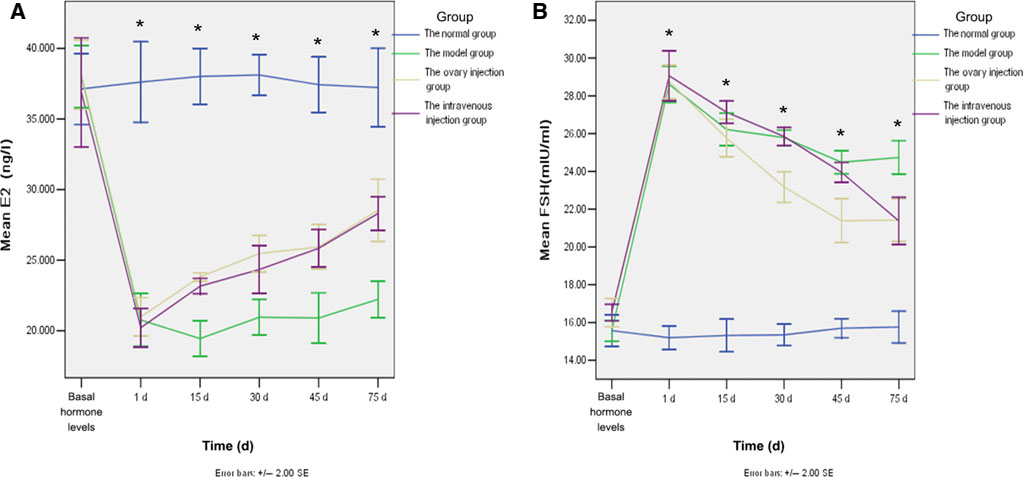
Levels of E_2_ (A) and FSH (B) were detected in blood samples drawn 1, 15, 30, 45 and 75 days after the induction of ovary damage was complete. At each of these time-points, blood samples were obtained from the tail vein of the following number of rats from each group: 25, 20, 15, 10 and 5 respectively; *P < 0.05.

For all four experimental rat groups, E_2_ and FSH levels in diestrus (the basal concentration) were determined. No significant differences between the groups were observed before injecting CTX. When the rat models of chemotherapy-induced ovarian damage were established, significant differences were observed. Levels of E_2_ decreased significantly, while levels of FSH increased significantly, compared with the normal group. These results suggested that ovarian function was seriously affected. After the rat models of chemotherapy-induced ovarian damage were established for 1–75 days (the HUMSCs transplantation in the ovary injection group and the tail vein injection group was finished for 1–75 days), the ovary injection group and the tail vein injection group exhibited higher level of E_2_ and lower levels of FSH, respectively, compared to the model group (Fig.3; *P* < 0.05), suggesting the HUMSCs transplantation can recover the ovary function. However, there was no statistically significant difference between the ovary injection group and the tail vein injection group on the levels of E_2_ and FSH at each time-point.

#### Fertility

**Figure 4:**
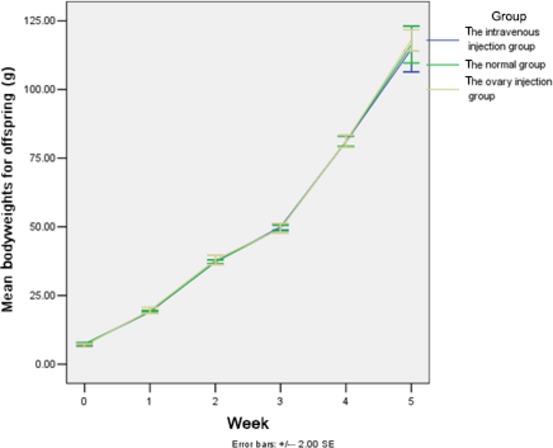
Bodyweights for offspring of the normal group, the ovary injection group and the tail vein injection group. The model group did not produce any offspring. Data are presented as the mean ± SD (five rats from each group).

We compared the birth start point of each group, the interval time of each birth, the number of each litter and the number of abnormality in each litter. All of the rats in the control group had normal reproduction with a 100% fertility rate. The average number of offspring in each litter was 5.71 ± 1.58, and the birth interval was 41.86 ± 13.92 days. For one rat that underwent ovarian injections, she produced offspring 49 days after caging (*e.g*. 64 days post-transplantation), and then had a second set of offspring (*n* = 3) during the observation period. For this rat, her fertility rate was 80% and the last delivery was 176 days after caging. Moreover, the average number in each litter was 3.14 ± 1.07 and the birth interval was 87.00 ± 50.54 days. For the intravenous injection group, one rat gave birth 60 days after caging (*e.g*. 75 days post-transplantation), and the other four rats delivered pups delivery during the observation period. Therefore, the fertility rate was 100% and the last delivery took place 167 days after caging. The average number in each litter was 3.42 ± 0.53, and the birth interval was 100.28 ± 41.31 days. None of the rats in the model group delivered pups during the observation period. Therefore, compared with the control group, the litter size of the ovarian injection group and the tail vein injection group decreased significantly (*P* = 0.000). In contrast, the litter size of the tail vein injection group was higher than that of ovary injection group (*P* = 0.000). Furthermore, there were no obvious differences in the general characteristics and growth of these offspring compared with those of the control group, as the offspring were found to have a normal reproductive capacity (Fig.4).

## Discussion

There have been a few studies published on the use of mesenchymal stem cells for the treatment of chemotherapy-induced premature ovarian failure. For example, Wang *et al*. found that HUMSCs transplantation reduced CTX-induced apoptosis of mouse ovarian cells and restored ovarian oestrogen secretion 18. These results suggested that this technique could treat ovary injury because of chemotherapy. In other studies 19–21, mesenchymal stem cells were derived from adipose seed cells and amniotic fluid to treat ovarian injury because of chemotherapy. More recently the use of umbilical cord mesenchymal stem cells for xenotransplantation has been considered. To address concerns regarding immunological rejection, HUMSC transplantation was performed in the present study to better understand the impact of these cells on the immunology of normal rats. The experimental results obtained demonstrate that rats have a natural tolerance for the transplantation of HUMSCs. Furthermore, even heterogeneous healthy individuals were found to tolerate HUMSCs, thereby facilitating their therapeutic effect 22. These results may be consistent with the low immunogenicity of HUMSCs, as demonstrated by the negative labelling of HUMSCs by HLA-DR in flow cytometry experiments 23. To ensure objectivity in the present study, additional scientific experiments were performed to avoid immune factors that were masked as studied factors.

Previously, CTX has been shown to reduce the number of ovarian follicles 18,19, and the present results confirm this observation. However, the effects of CTX were also found to differ according to the follicle stage. For example, when injections of CTX were made on day 15, the model group, the ovary injection group and the intravenous injection group showed no significant difference in their primary follicles compared with the normal group. However, for the same three groups, there were fewer secondary follicles and antral follicles compared with the normal group, indicating that CTX destroys ovarian secondary preantral and antral follicles first. After a longer period of time, the model group, ovary injection group and the intravenous injection group exhibited a statistically significant reduction in the number of primary follicles compared with the normal group, and the follicles were gradually consumed. However, in the ovarian injection group and the tail vein injection group, the number of secondary follicles and antral follicles were higher than those in the model group, indicating that antiapoptotic effect of HUMSCs primarily manifests in the protection of secondary follicles and antral follicles. Transplanted HUMSCs can secret anti-apoptosis related factors, such as VEGF, IGF-1 and HGF. These cell factors have functions in anti-apoptosis, promoting the generation of blood vessels and inhibiting the apoptosis of ovary follicles 11. Therefore, transplanted HUMSCs could serve to reduce, or even repair CTX-mediated damage of mature follicles and partially restore rat ovarian endocrine and reproductive functions.

Although the use of mesenchymal stem cells for the treatment of CTX-induced ovarian injury has been described, the transplantation methods have differed. In particular, local ovarian transplantation 11 and intravenous transplantation 18 have been reported. A goal of the present study was to optimize the efficacy of HUMSC transplantation as a minimally invasive technique. Both the local injection of HUMSCs and the tail vein injection of HUMSCs were accompanied by a rapid recovery of hormone levels. Regarding the former technique, HUMSCs aggregation in local regions of the ovary is possible. However, anti-apoptotic factors may be secreted by HUMSCs, and may also be present at higher concentrations proximal to the ovary. For treatment of the ovary injection group and the tail vein injection group, the long-term effects on ovary function were similar, although the administration of HUMSCs *via* tail vein resulted in the distribution of HUMSCs among multiple organs of the rat. This may represent an advantage of this transplantation approach as chemotherapy can damage many organs, including liver, kidney and urocyst. Thus, the injection of HUMSCs intravenously may contribute to the repair of multiple organs by a ‘boomerang’ effect. Another consideration regarding the two methods of HUMSC administration was the invasive nature of the ovary injection that required opening of the abdomen, *versus* tail vein transplantation. The latter represents a more minimally invasive method that can cause less damage and involve a shorter recovery time. The latter method also provides the opportunity for multiple treatments to be administered and various concentrations of HUMSCs to be applied. Thus, tail vein injections for animal models, and intravenous injections in the clinic, may be a preferred method of HUMSC administration compared with local ovary injection.

For tail vein injections, cells must be adequately dissociated to prevent the formation of cell aggregates and thrombi and to prevent serious complications. In the present study, thrombi did form (Fig. S6), and this may be because of the smaller size of the veins in rats and the larger size of the HUMSCs. However, it is has been suggested that if HUMSCs were resuspended for clinical application, the potential for thrombus formation would be less.

Another key aspect of the present study was the restoration of fertility in a subset of rats that underwent transplantation. For these rats, healthy offspring were produced, thereby indicating that HUMSCs not only contributed to the recovery of endocrine function in the ovary but also improved ovarian oviposit function. In addition, there was no obvious difference between the offspring of transplanted rats and the controls, or between the sexual maturation rates of the transplanted rats *versus* controls. Based on these results, the transplantation of HUMSCs may not affect the growth and development of offspring in humans, although this remains to be confirmed.

## Supporting Information

**Figure S1** Representative pictures of the tail vein injections (A) and ovary injections (B) that were performed.

**Figure S2** The structures of the ovary follicle types observed.

**Figure S3** Successful isolation of HUMSCs.

**Figure S4** (A) HUMSC growth curve. (B) Detection of apoptosis by flow cytometry. (C) Detection of cell cycle distribution by flow cytometry.

**Figure S5** (a1) PKH26-labelled HUMSCs under light microscopy. (a2) PKH26-labelled HUMSCs under fluorescence microscopy.

**Figure S6** TTC staining of a brain section from a rat exhibiting hemiplegia following a tail vein injection of HUMSCs.

**Figure S7** HE staining of pathological sections from various organs.

**Figure S8** Distribution of labelled HUMSCs in ovary tissues obtained from the ovary injection group and detected with light microscopy (a1) and fluorescence microscopy (a2). These tissues were compared with tissues obtained near the end of the uterus, which were observed with light microscopy (b1) and fluorescence microscopy (b2).

**Figure S9** Distribution of labelled HUMSCs in ovary (a), uterus (b), liver (c) and kidney (d) tissues obtain from the tail vein injection group that were observed under light microscopy and fluorescence microscopy (designated 1 and 2 respectively).
