# Supplementary figures and images for: Human umbilical cord mesenchymal stem cell transplantation restores damaged ovaries
Source: J Cell Mol Med. 2015 Apr 29;19(9):2108–17. doi: 10.1111/jcmm.12571 (PMC4568915; doi:10.1111/jcmm.12571)

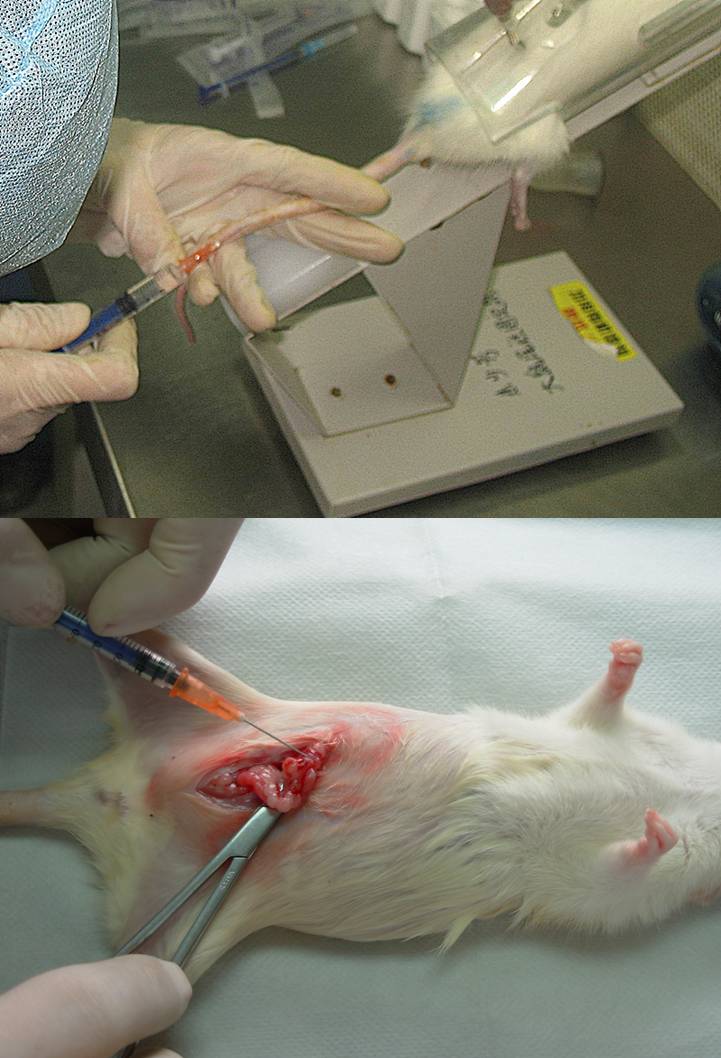

Supplement: Supplementary file 1 [file jcmm0019-2108-sd1.jpg]

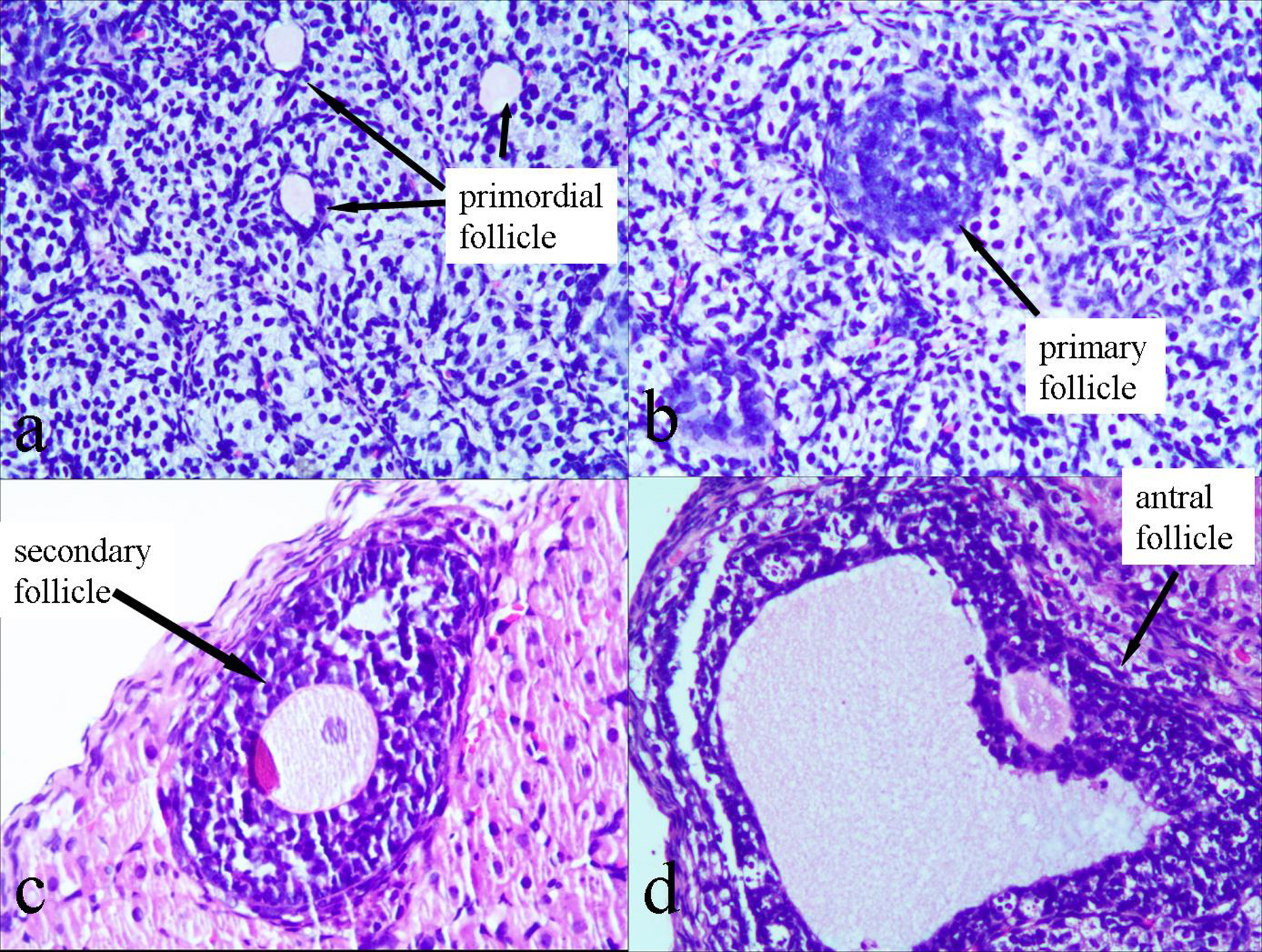

Supplement: Supplementary file 2 [file jcmm0019-2108-sd2.jpg]

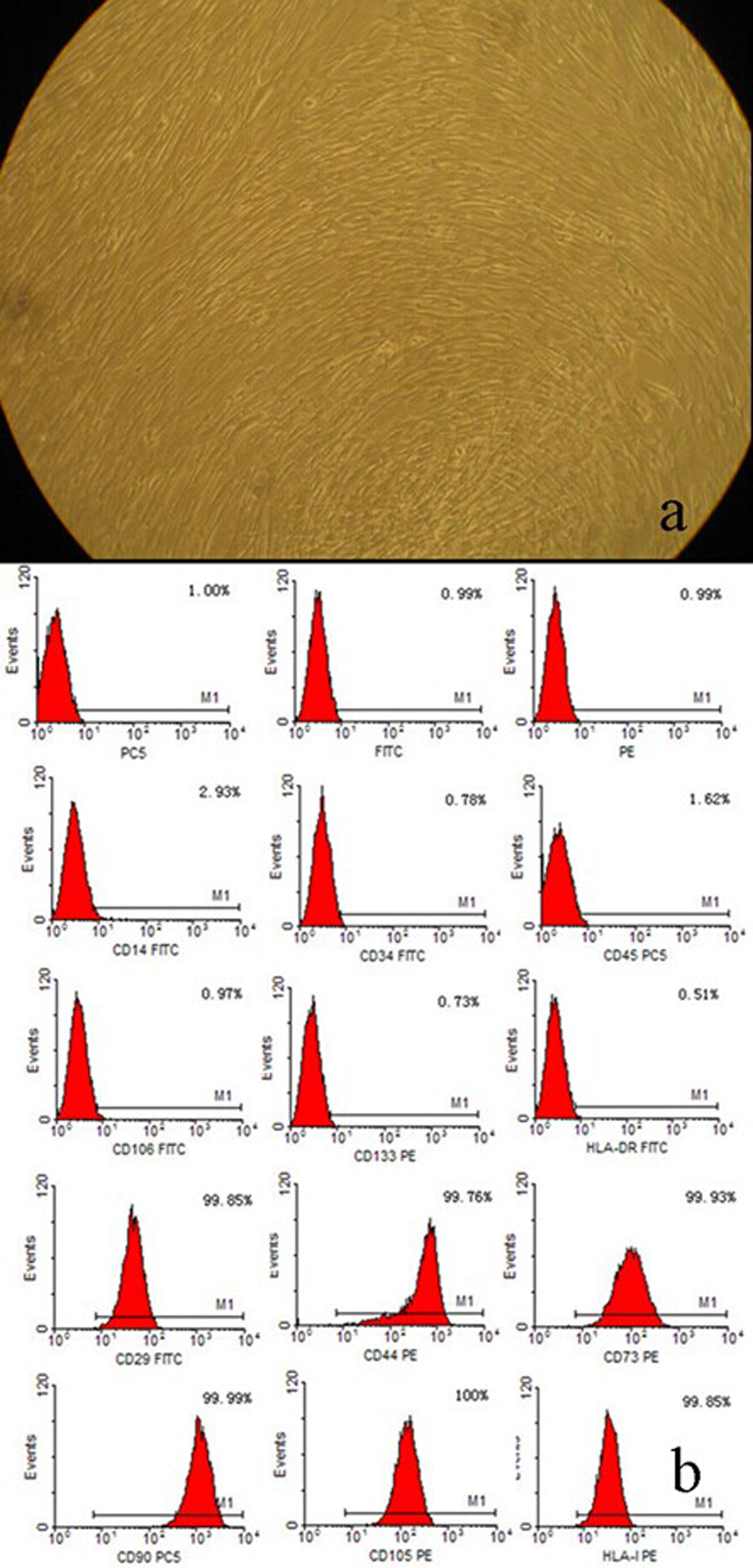

Supplement: Supplementary file 3 [file jcmm0019-2108-sd3.jpg]

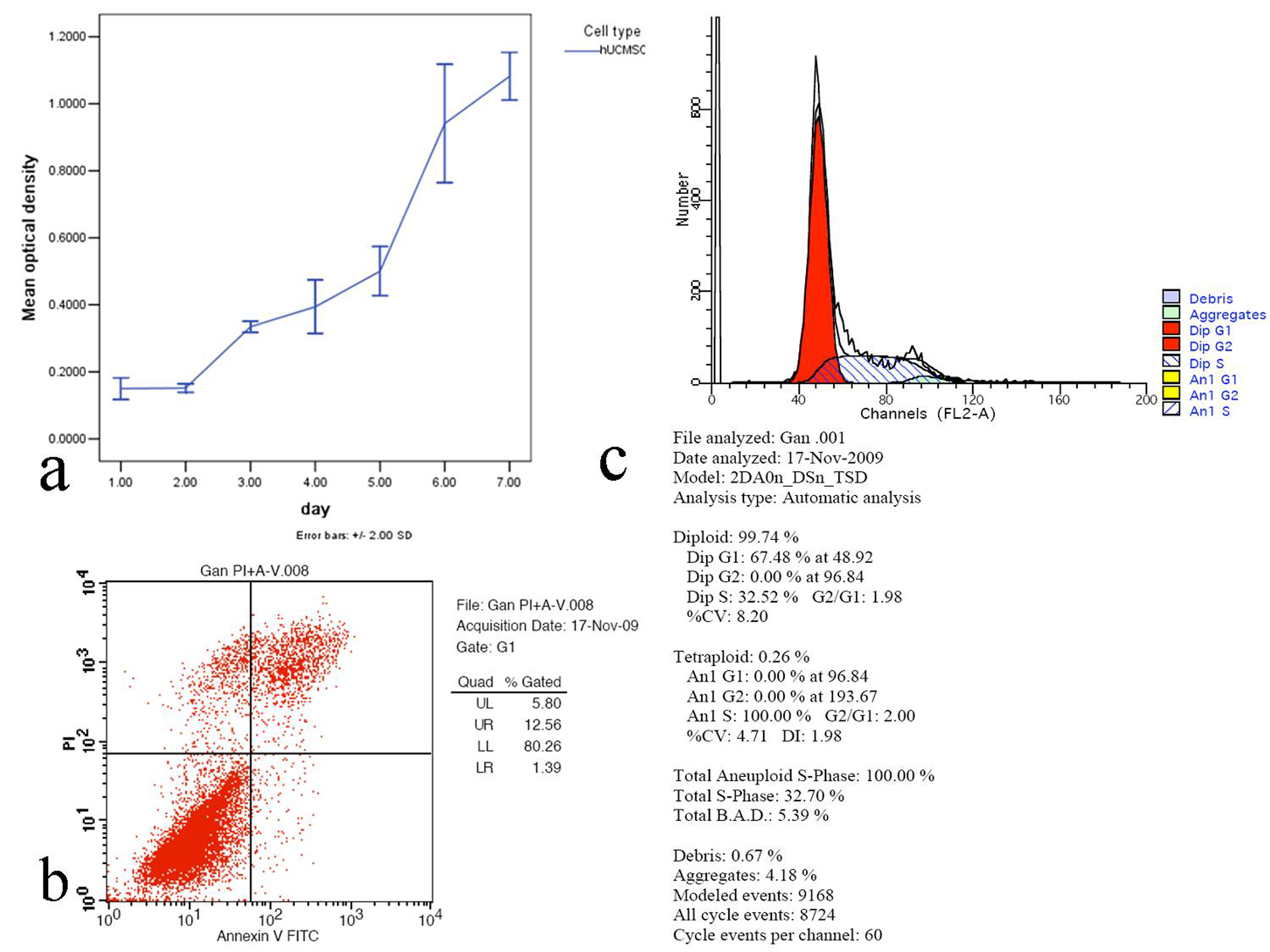

Supplement: Supplementary file 4 [file jcmm0019-2108-sd4.jpg]

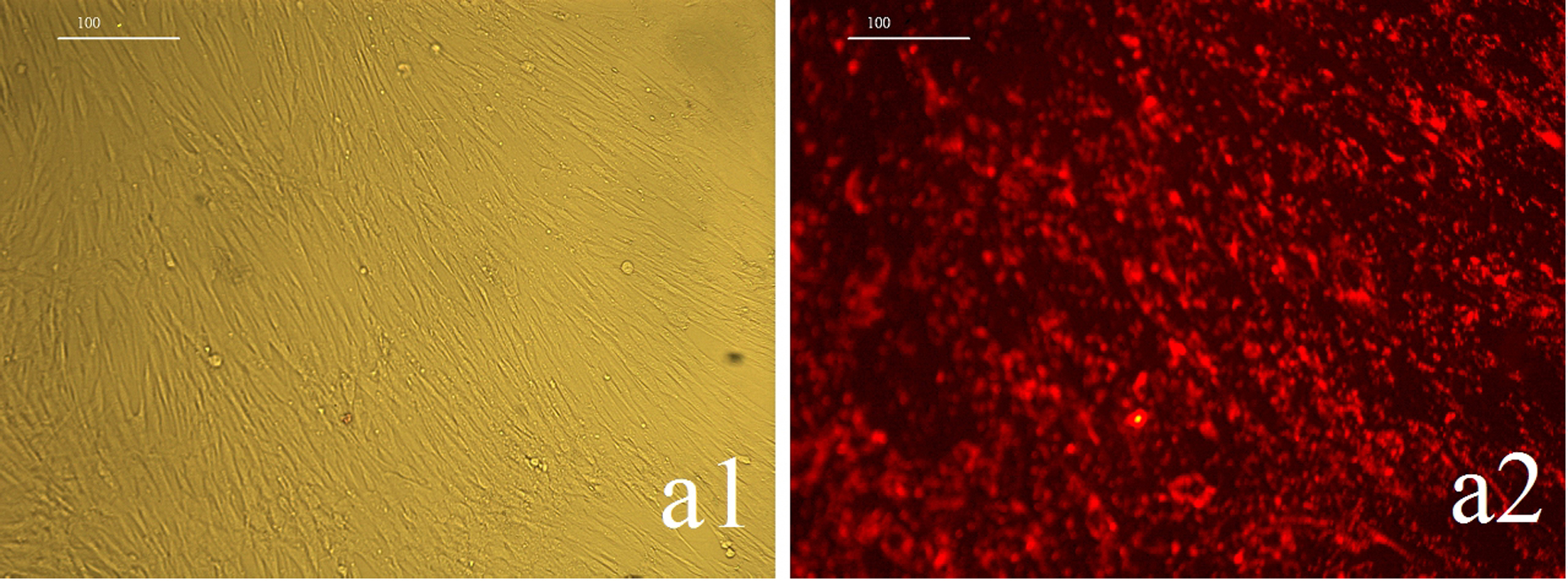

Supplement: Supplementary file 5 [file jcmm0019-2108-sd5.jpg]

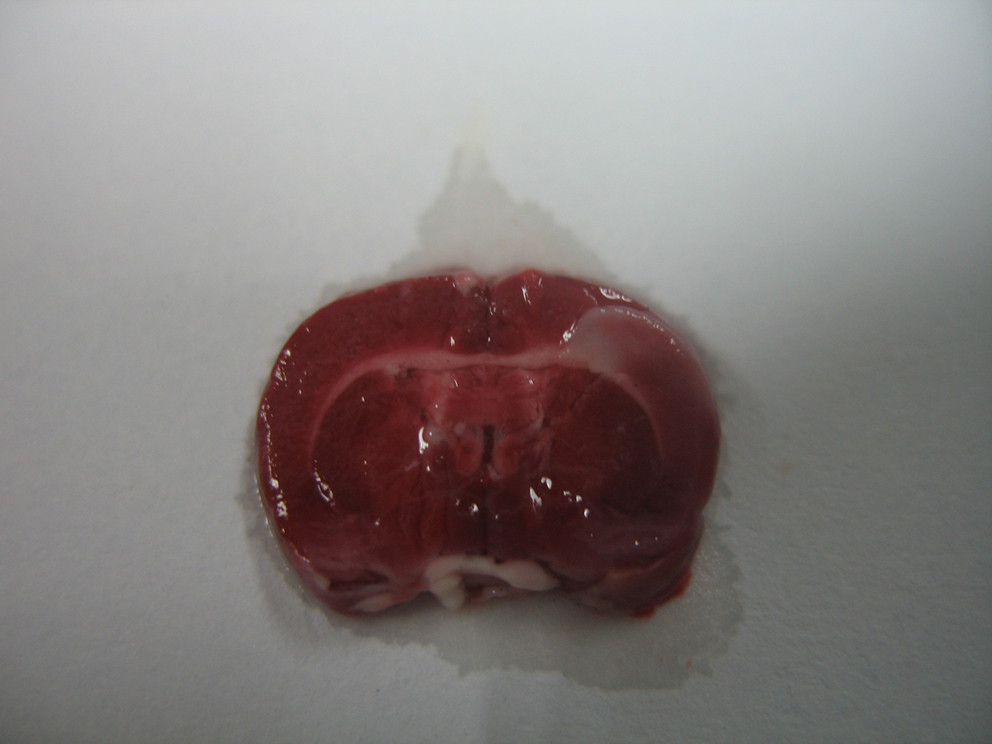

Supplement: Supplementary file 6 [file jcmm0019-2108-sd6.jpg]

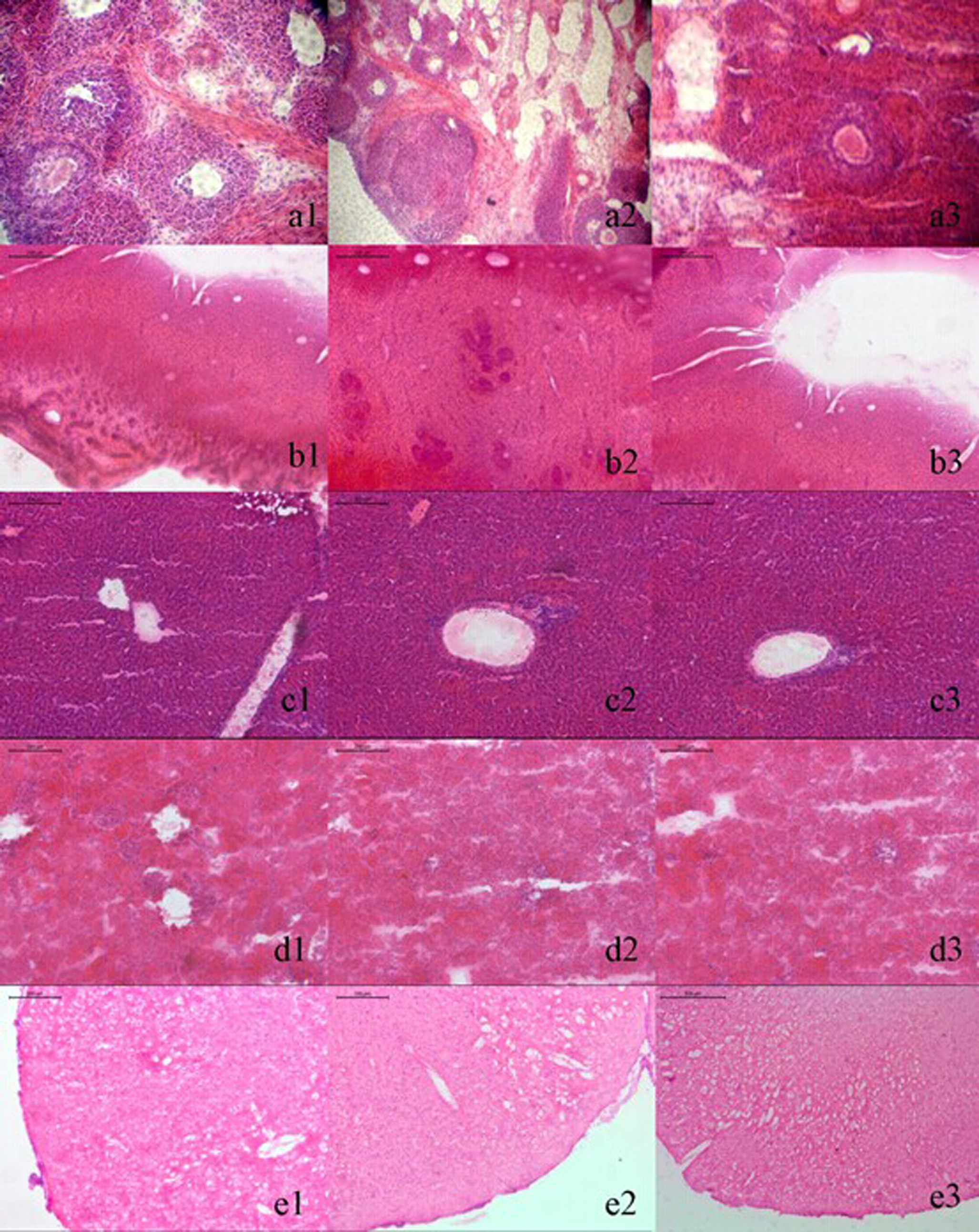

Supplement: Supplementary file 7 [file jcmm0019-2108-sd7.jpg]

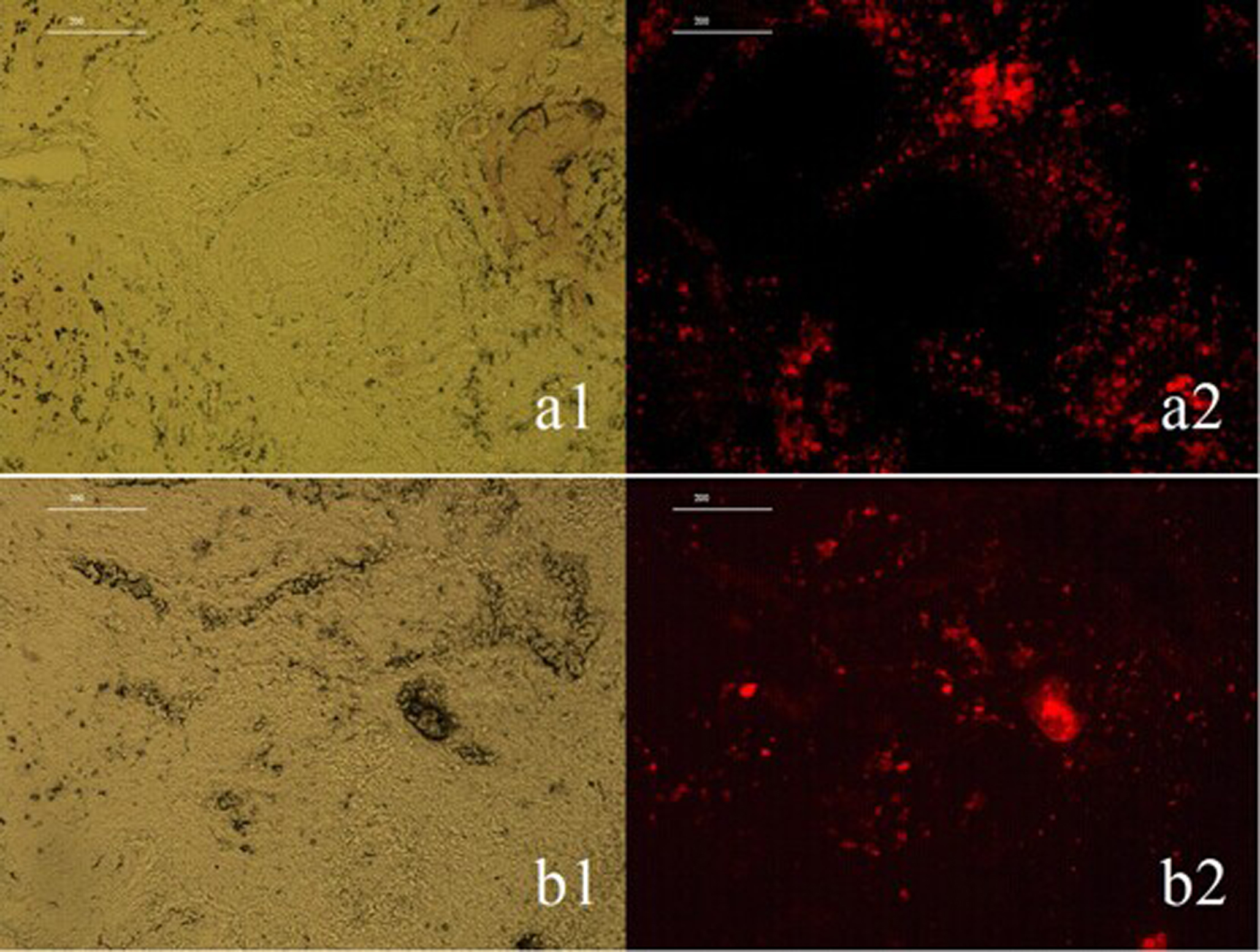

Supplement: Supplementary file 8 [file jcmm0019-2108-sd8.jpg]

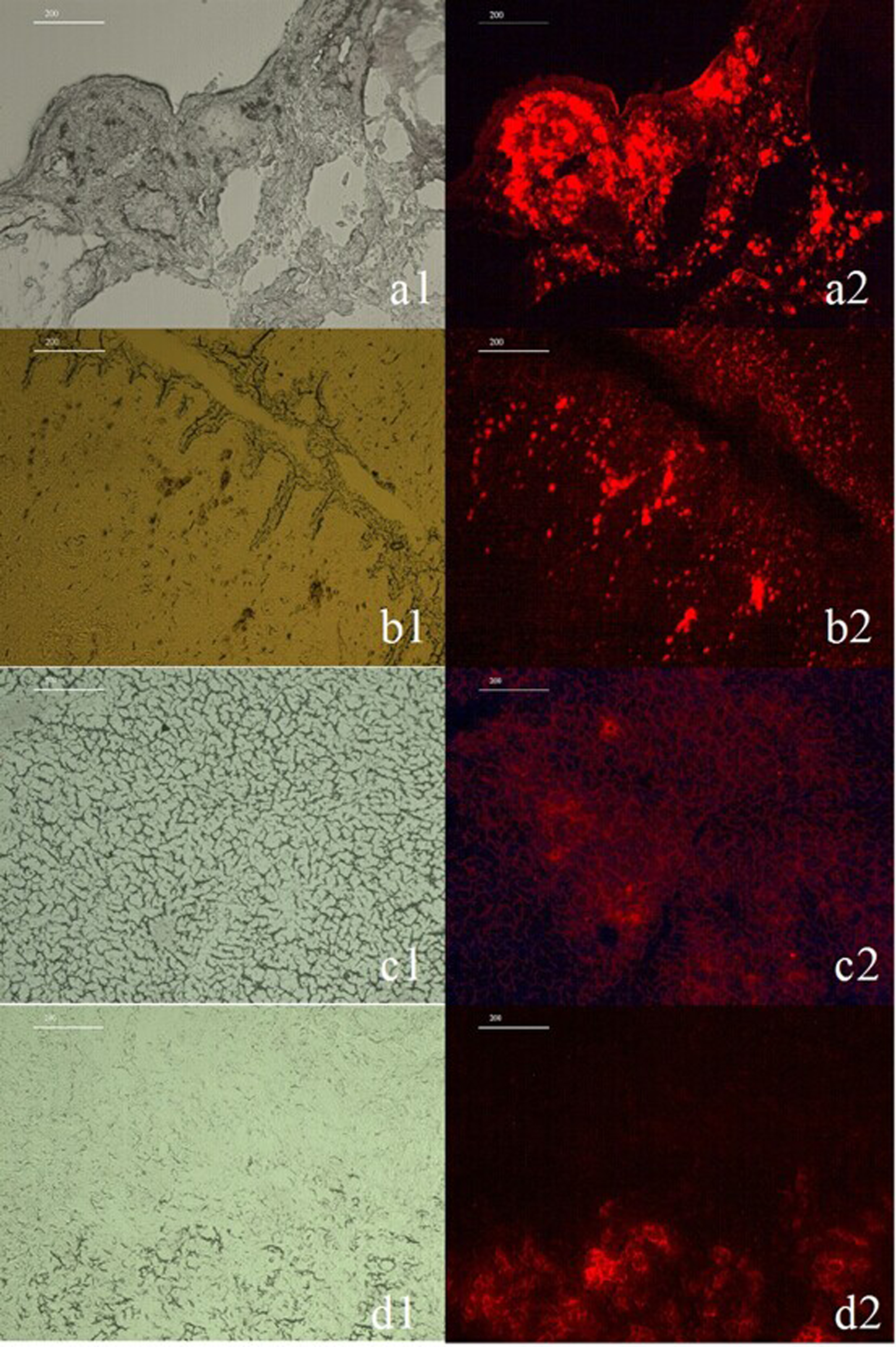

Supplement: Supplementary file 9 [file jcmm0019-2108-sd9.jpg]
